# Evaluating the role of follicular output rate in clinical and embryological outcomes in IVF: a prospective study

**DOI:** 10.1007/s00404-026-08487-z

**Published:** 2026-06-10

**Authors:** Arshiya Firdaus, Anjali S. Mundkur, Vidyashree G. Poojari, Pratap Kumar, Srisailesh Vitthala, Prashanth K. Adiga

**Affiliations:** 1 Department of Reproductive Medicine and Surgery, Kasturba Medical College Manipal, Manipal Academy of Higher Education, Manipal, Karnataka 576104 India; 2 Department of Reproductive Medicine and Surgery, MMC IVF Center, Dubai, United Arab Emirates

**Keywords:** Follicular output rate, IVF, Ovarian response, Clinical pregnancy, Live birth, PCOS, ART, Ovarian reserve

## Abstract

**Purpose:**

To examine the association between follicular output rate (FORT) and embryological and clinical outcomes, including live birth rate, in patients undergoing in vitro fertilization (IVF).

**Methods:**

This prospective cohort study enrolled 166 patients undergoing IVF between October 2023 and January 2025. FORT was calculated as the ratio of pre-ovulatory follicles (16–24 mm) on trigger day to baseline antral follicle count (AFC; 3–9 mm), multiplied by 100. Patients were stratified into low, medium, and high FORT tertiles, and FORT was also evaluated as a continuous variable in multivariable logistic regression. An exploratory receiver operating characteristic (ROC) analysis was performed to assess discriminatory performance and to identify a data-derived reference threshold. All analyses were conducted separately for non-PCOS and PCOS cohorts.

**Results:**

In non-PCOS patients (n = 136), the number of oocytes retrieved, mature oocytes, and good-quality embryos differed significantly across FORT tertiles (all p < 0.05). Clinical pregnancy rates were 14.9%, 27.8%, and 37.2% in the low, medium, and high FORT groups respectively (p = 0.045), with corresponding differences in live birth rates (p = 0.049). On multivariable analysis, FORT (OR 1.04; 95% CI 1.002–1.079; p = 0.039) and the number of available embryos (OR 1.35; 95% CI 1.082–1.676; p = 0.008) remained independently associated with clinical pregnancy. Exploratory ROC analysis yielded an AUC of 0.607 (95% CI 0.52–0.69). A data-derived reference threshold of FORT at 64.2% was associated with higher clinical pregnancy rates compared with lower FORT values (40.9% vs. 23.3%; p = 0.030; OR 2.27, 95% CI 1.14–4.55). No significant associations were observed in the PCOS subgroup, though this analysis was limited by small sample size (n = 30).

**Conclusions:**

FORT is significantly associated with embryological and clinical pregnancy outcomes, including live birth, in non-PCOS IVF patients. As a cycle-specific functional measure of follicular responsiveness, FORT may complement static ovarian reserve markers for counselling and outcome stratification. FORT values in the higher range (approximately ≥ 65%) were associated with improved clinical pregnancy rates, but the limited discriminatory performance of the ROC analysis underscores the need for external prospective validation of this cutoff before any clinical implementation.

## What does this study adds to the clinical work

**Table:** 

FORT offers complementary, cycle-specific insight into ovarian responsiveness and is associated with key IVF clinicaloutcomes. When used alongside established markers such as AMH and AFC, it may enhance personalized counsellingand outcome stratification	

## Introduction

Assisted reproductive technology (ART) has transformed the management of infertility worldwide. However, accurate prediction of ovarian response to controlled ovarian stimulation remains a key challenge in clinical practice [1, 2]. At present, treatment planning is largely guided by static indicators of ovarian reserve, primarily anti-Müllerian hormone (AMH) levels and antral follicle count (AFC). While these parameters estimate the size of the follicular pool, they do not adequately reflect the ovary’s functional response to exogenous gonadotropins [3, 4]. A marker that integrates baseline reserve with functional responsiveness would therefore represent a meaningful advance in cycle management.

The follicular output rate (FORT) was introduced by Genro et al. in 2011 as a novel measure of follicular responsiveness [5]. It is defined as the ratio of the number of pre-ovulatory follicles (measuring 16–24 mm on trigger day) to the baseline AFC (3–9 mm measured on cycle days 2–3), multiplied by 100. By expressing pre-ovulatory follicle development relative to the available baseline pool, FORT provides a cycle-specific index of functional ovarian responsiveness that complements, rather than replaces, static reserve markers [5–9]. In the present study, FORT was calculated using the original definition proposed by Genro et al., with a denominator restricted to 3–9 mm follicles, to ensure consistency with prior reports.

Despite its theoretical rationale, clinical evidence supporting FORT's utility remains limited. Published studies have focused on patients undergoing long GnRH agonist protocols, leaving generalizability to antagonist and short flare-agonist regimens uncertain [5, 6, 10, 11]. Reported associations between FORT and clinical outcomes including clinical pregnancy and live birth are inconsistent, and no consensus threshold for clinical stratification has been established. Previous studies have relied on arbitrary tertile boundaries rather than empirically derived cut-points, restricting the translation of research findings into practice [5, 6, 10, 12, 13]. Additionally, few studies have included live birth as a primary endpoint; most have focused on cycle-level surrogates.

The present study was designed to address these gaps. We prospectively evaluated the association between FORT and a comprehensive set of embryological and clinical outcomes including live birth in a cohort undergoing both antagonist and short flare-agonist protocols. Secondary objectives included multivariable identification of independent predictors of clinical pregnancy and an exploratory ROC analysis to derive a data-based FORT threshold that may guide stratification and counselling.

## Materials and methods

### Study design and setting

This prospective cohort study was conducted at the Department of Reproductive Medicine and Surgery, Kasturba Medical College, Manipal Academy of Higher Education (MAHE), Manipal, India. The study received approval from the Institutional Review Board (IEC1: 190/2023; approval date 08 August 2023) and was prospectively registered with the Clinical Trials Registry of India (CTRI/2023/10/058192) prior to enrolment. All participants provided written informed consent.

### Study population

#### Inclusion criteria

Women aged below 40 years undergoing their first IVF cycle between October 2023 and January 2025 were eligible.

#### Exclusion criteria

Patients with adenomyosis, moderate-to-severe male factor infertility, autoimmune disease, significant uterine pathology (submucous fibroids, intrauterine adhesions), or large endometriomas that precluded reliable AFC assessment were excluded.

### Ovarian stimulation protocols

Stimulation commenced on cycle day 2 or 3. Two standardized protocols were used, with selection individualized according to patient characteristics:

### Flexible GnRH antagonist protocol

Gonadotropins were initiated at 150–450 IU/day. A GnRH antagonist (cetrorelix 0.25 mg/day) was introduced on stimulation day 5 or 6 when a lead follicle reached ≥ 14 mm or serum estradiol exceeded 400 pg/mL, and continued until trigger day.

### GnRH agonist short flare protocol

Leuprolide acetate 80 µg/day was commenced on day 2 or 3 of the cycle; gonadotropins were added two days later and continued until trigger day.

Final oocyte maturation was triggered when ≥ 3 follicles reached ≥ 18 mm, using either recombinant hCG 250 µg subcutaneously or leuprolide acetate 1 mg subcutaneously (in cycles at elevated OHSS risk). Oocyte retrieval was performed 36 h after triggering under transvaginal ultrasound guidance.

### FORT calculation and patient stratification

Transvaginal ultrasound examinations were performed using an 8.0 MHz probe. AFC was assessed on cycle day 2 or 3 by counting all follicles measuring 3–9 mm across both ovaries. The pre-ovulatory follicle count (PFC) was determined on trigger day, including all follicles measuring 16–24 mm bilaterally. FORT was calculated as:

$${\text{FORT }}\left( \% \right) = \left( {{\mathrm{PFC}}\,{\mathrm{on}}\,{\mathrm{trigger}}\,{\mathrm{day}}/{\mathrm{AFC}}\,{\mathrm{at}}\,{\mathrm{baseline}}} \right) \times 100$$

All AFC and PFC measurements were performed by the same two experienced sonographers to minimise inter-observer variability. For the between-group comparisons and clinical outcome analyses, patients were stratified into tertiles based on their study-specific FORT distribution: low FORT (< 33rd percentile), medium FORT (33rd–67th percentile), and high FORT (> 67th percentile). FORT was additionally analysed as a continuous variable in multivariable regression. These tertile boundaries were derived empirically from the study cohort distribution and differ from the generic one-third divisions used in some prior publications [5, 6, 12], a distinction that should be borne in mind when making cross-study comparisons.

### Embryo transfer protocol

All patients underwent frozen embryo transfer (FET) using a hormone replacement therapy (HRT) protocol. Pituitary downregulation was achieved with a depot GnRH agonist (leuprolide acetate 3.75 mg intramuscularly) administered on day 21 of the preceding cycle. Endometrial preparation was performed with estradiol valerate 2 mg orally three times daily; progesterone supplementation (vaginal progesterone gel twice daily combined with oral dydrogesterone 20 mg twice daily) was initiated when endometrial thickness reached ≥ 7 mm. Two cleavage-stage frozen embryos were transferred per cycle. Luteal phase support was continued until 10 weeks' gestation in patients who achieved pregnancy.

Only the first embryo transfer was analysed. This approach was determined by the study recruitment period (October 2023 to January 2025) and by follow-up patterns observed in our routine practice. A subset of patients who did not achieve pregnancy after the initial embryo transfer did not proceed with additional cycles within the available follow-up, which would have introduced substantial attrition bias if cumulative transfer outcomes had been analyzed. For this reason, outcome assessment was restricted to the first embryo transfer. This restriction represents a study limitation, as cumulative reproductive outcomes may be underestimated, particularly among patients with higher FORT values and a greater number of available embryos. Evaluation of cumulative outcomes across all transfers will be important in future studies with extended follow-up durations.

#### Rationale for HRT-FET protocol

All transfers were conducted in HRT-FET cycles. While this standardises the endometrial preparation and reduces variability in cycle monitoring, it also means that findings may not be directly generalisable to natural-cycle or modified natural-cycle FET. The potential influence of the exogenous hormonal environment on endometrial receptivity and FORT-outcome associations is acknowledged.

### Outcome measures

#### Primary outcomes (embryological)

Number of oocytes retrieved, number of mature (MII) oocytes, number of good-quality embryos (grades 1 and 2 per the Istanbul consensus grading criteria).

#### Secondary outcomes (clinical)

Clinical pregnancy rate: defined as the presence of at least one intrauterine gestational sac with fetal cardiac activity at 6–7 weeks' gestation per embryo transfer cycle, in accordance with the ICMART-WHO glossary [14].

Live birth rate: defined as the delivery of at least one live infant beyond 24 weeks' gestation per embryo transfer cycle [14].

### Patient flow and analytical subgroups

Of the 166 patients enrolled, 136 were non-PCOS and 30 had PCOS diagnosed according to the revised Rotterdam criteria (2003). All 166 patients were included in embryological outcome analyses. Of these, 156 proceeded to embryo transfer; 10 patients were excluded from clinical outcome analyses (natural conception n = 5; empty follicle syndrome n = 2; embryo disintegration n = 1; failed embryo formation n = 2). Given the substantially elevated AFC and AMH values in the PCOS subgroup relative to non-PCOS patients, all between-group outcome analyses were conducted separately within each subgroup to minimise confounding from the differing baseline ovarian reserve profiles.

### Statistical analysis

Statistical analyses were performed using Python (version 3.12, Jupyter Notebook environment). Data distributions were examined by visual inspection of histograms and by formal assessment with HistoFit software. Continuous variables with normal distributions are presented as mean ± standard deviation, whereas non-normally distributed variables are presented as median and interquartile range (IQR). Categorical variables are reported as counts and percentages. Comparisons across FORT tertiles were performed using the Kruskal–Walli’s test for continuous variables and the chi-square test for categorical variables. For analyses comparing two groups (pregnant vs. non-pregnant), the Mann–Whitney U test was applied. Multivariable logistic regression was used to identify independent predictors of clinical pregnancy. Covariates included in the model were: age, BMI, AFC, AMH, PFC, FORT (continuous), total gonadotropin dose, number of oocytes retrieved, number of M2 oocytes, and number of available embryos. FORT was entered as a continuous variable to evaluate its contribution independently of the tertile classification.

Tertile stratification was used to characterise clinical trends across the FORT distribution and to facilitate comparison with prior publications that have also employed tertile groupings [5, 6, 12]. The continuous-variable multivariable analysis was used to assess independent predictive value while adjusting for confounders, which is the more analytically rigorous approach for a continuous marker. The two analyses are complementary rather than redundant: tertile-based comparisons describe the clinical gradient in outcomes, while the regression analysis establishes FORT's independent contribution.

#### ROC analysis

Receiver operating characteristic analysis was performed as an exploratory secondary analysis to assess the discriminatory ability of FORT for clinical pregnancy and to identify a data-driven reference threshold for hypothesis generation. Statistical significance was set at p < 0.05.

## Results

### Study population and baseline characteristics

Table 1 presents the baseline characteristics of the full study population (n = 166). The median age was 33 years (IQR 29.5–36). Primary infertility was the indication in 135 patients (81.3%). The GnRH antagonist protocol was used in 134 patients (80.7%) and the short flare-agonist protocol in 32 (19.3%). Unexplained infertility was the most frequent diagnosis (n = 61, 36.7%), followed by tubal factor (n = 39, 23.5%), PCOS (n = 30, 18.1%), mild male factor (n = 18, 10.8%), and endometriosis (n = 18, 10.8%). Among the 156 patients who reached embryo transfer, clinical pregnancy was achieved in 48 (30.8%) and live birth in 46 (29.5%).

**Table 1:** Baseline characteristics of the study population (n = 166)

Parameter	Value (n = 166)
Age (years)	33 (29.5–36)
BMI (kg/m^2^)	23.5 (22.15–25.5)
Duration of infertility (years)	5 (3–7)
AMH (ng/mL)	1.84 (1.25–2.46)
Total gonadotropin dose (IU)	3200 (2497–3750)
Primary infertility	135 (81.3%)
Secondary infertility	31 (18.7%)
Antagonist protocol	134 (80.7%)
Short flare-agonist protocol	32 (19.3%)
PCOS patients	30 (18.1%)
Mild male factor infertility	18 (10.8%)
Tubal factor infertility	39 (23.5%)
Unexplained infertility	61 (36.7%)
Endometriosis	18 (10.8%)
β-hCG positive (per transfer)	56/156 (35.9%)
Clinical pregnancy rate (per transfer)	48/156 (30.8%)
Live birth rate (per transfer)	46/156 (29.5%)

Data expressed as median (IQR) for continuous variables and n (%) for categorical variables

*AMH* anti-Müllerian hormone, *BMI* body mass index, *PCOS* polycystic ovary syndrome

### Embryological and clinical outcomes by FORT tertile: non-PCOS cohort

Table 2 presents the embryological and clinical outcomes across FORT tertiles in the non-PCOS cohort (n = 136). Median FORT values were 40.8% (IQR 36.0–46.0%), 61.0% (IQR 57.3–63.4%), and 83.0% (IQR 75.0–85.0%) in the low, medium, and high FORT tertiles, respectively. Baseline characteristics, including age, BMI, infertility duration, stimulation protocol, and total gonadotropin dose, did not differ significantly across tertiles.

**Table 2:** Clinical and embryological outcomes across FORT Tertiles: non-PCOS cohort (n = 136)

Parameter	Low FORT < 33rd percentile (n = 52) 15.7–50%	Medium FORT 33rd–67th percentile (n = 39) 52.4–70.6%	High FORT > 67th percentile (n = 45) 71.4–100%	p
Age (years)	33 (29–36.25)	33.5 (29.25–36)	34 (30–36)	0.885
BMI (kg/m^2^)	23.85 (22.5–25.55)	23.25 (22.02–25)	24 (22.4–26)	0.521
Infertility duration (years)	5 (3–7.25)	5 (3–7)	5 (4–9)	0.294
AMH (ng/mL)	1.56 (0.99–2.06)	2.3 (1.67–3.46)	1.8 (1.23–2.36)	0.002*
AFC	11 (9–15.25)	15.5 (10.25–21)	10 (8–13)	< 0.05*
Short flare protocol (n)	12	10	10	0.822
Total gonadotropin dose (IU)	3450 (2700–3750)	3000 (2175–3600)	3600 (2700–3900)	0.119
PFC (16–24 mm)	5 (3–6.25)	9.5 (7–12.5)	9 (6–11)	< 0.05*
FORT (%)	40.8 (36.0–46.0)	61.0 (57.3–63.4)	83.0 (75.0–85.0)	< 0.05*
Oocytes retrieved	5 (4–7)	8.5 (7–12.75)	8 (6–11)	< 0.05*
Mature (MII) oocytes	4 (3–6)	8 (6–11)	7 (5–9)	< 0.05*
Good-quality embryos (gr. 1 + 2)	3 (2–4)	4.5 (3–6)	4 (3–6)	< 0.05*
Clinical Outcomes (n = 126; low n = 47, medium n = 36, high n = 43)				
β-hCG positive	9 (19.1%)	11 (30.6%)	17 (39.5%)	0.104
Clinical pregnancy	7 (14.9%)	10 (27.8%)	16 (37.2%)	0.045*
Live birth	7 (14.9%)	9 (25.0%)	16 (37.2%)	0.049*

*AFC* antral follicle count, *FORT* follicular output rate, *PFC* pre-ovulatory follicle count, *gr.* grade

*p < 0.05 (Kruskal–Wallis test for continuous variables; chi-square test for categorical outcomes)

Oocyte yield, number of mature (MII) oocytes, pre-ovulatory follicle count, and number of good-quality embryos were significantly higher across FORT tertiles (all p < 0.05; Table 2).

Clinical pregnancy rates were 14.9%, 27.8%, and 37.2% in the low, medium, and high FORT tertiles, respectively (p = 0.045). Corresponding live birth rates were 14.9%, 25.0%, and 37.2% (p = 0.049).

### PCOS subgroup analysis

Table 3 presents outcomes in the PCOS subgroup (n = 30; 10 per tertile). No statistically significant differences were observed across tertiles for oocyte yield, number of mature oocytes, good-quality embryo count, clinical pregnancy, or live birth rates. Clinical pregnancy rates were 40%, 60%, and 50% in the low, medium, and high FORT tertiles, respectively (p = 0.67); however, the subgroup was small and underpowered for clinical outcome comparisons.

**Table 3:** Clinical and embryological outcomes across FORT tertiles: PCOS cohort (n = 30)

Parameter	Low FORT < 33rd percentile (n = 10) < 47%	Medium FORT 33rd–67th (n = 10) 47–70%	High FORT > 67th percentile (n = 10) > 70%	p
Age (years)	31.5 (31.0–33.0)	31.5 (29.25–33.0)	31.5 (29.25–33.0)	0.896
BMI (kg/m^2^)	22.15 (20.12–24.45)	23.65 (21.15–24.58)	22.75 (19.55–24.0)	0.924
Infertility duration (years)	4.0 (3.0–6.0)	6.0 (4.0–6.0)	4.5 (3.25–6.0)	0.703
AMH (ng/mL)	8.74 (5.3–11.4)	5.58 (5.14–6.47)	6.06 (4.97–8.86)	0.278
AFC	40 (32.5–40.0)	26 (25.0–33.75)	23 (19.25–25.0)	0.01*
Total gonadotropin dose (IU)	1987.5 (1575–2081.25)	2275 (2025–2325)	2137.5 (1743.75–2400)	0.412
PFC (16–24 mm)	15.5 (11.75–17.0)	18.0 (14.0–19.75)	17.5 (16.25–22.75)	0.285
FORT (%)	42.5 (36.66–42.5)	57.54 (53.97–67.66)	81.66 (75.57–86.1)	< 0.05*
Oocytes retrieved	17.5 (13.25–20.5)	17.5 (16.25–18.75)	15.5 (14.0–18.5)	0.863
Mature (MII) oocytes	11.0 (8.5–15.0)	14.5 (11.3–17.75)	14.0 (11.25–16.0)	0.387
Good-quality embryos	8.0 (6.5–10.75)	10.0 (8.0–11.75)	10.0 (5.0–11.0)	0.648
β-hCG positive	5 (50%)	7 (70%)	7 (70%)	0.56
Clinical pregnancy	4 (40%)	6 (60%)	5 (50%)	0.67
Live birth	4 (40%)	6 (60%)	5 (50%)	0.67

The PCOS subgroup was underpowered for clinical outcome analysis (n = 10 per tertile); null findings should not be interpreted as evidence of absence of effect. Kruskal–Wallis test for continuous variables; chi-square for categorical outcomes

*p < 0.05

### Comparison of pregnant and non-pregnant patients

Table 4 compares characteristics between pregnant (n = 48) and non-pregnant (n = 108) patients in the full transfer cohort. Pregnant patients had significantly higher AMH, AFC, PFC, FORT, oocyte yield, mature oocyte number, and available embryo count compared to non-pregnant patients (all p < 0.05). Age, BMI, and total gonadotropin dose did not differ significantly between the groups.

**Table 4:** Comparison of clinical characteristics between pregnant and non-pregnant groups

Parameter	Pregnant (n = 48)	Non-Pregnant (n = 108)	p
Age (years)	31.50 (30.00–34.25)	33.00 (29.00–36.00)	0.305
BMI (kg/m^2^)	23.40 (22.08–24.83)	23.60 (22.05–25.25)	0.703
AMH (ng/mL)	2.46 (1.84–4.61)	1.89 (1.35–3.53)	0.024*
AFC	18.50 (11.00–25.00)	13.00 (9.00–18.00)	0.010*
PFC	11.00 (7.00–15.50)	7.00 (5.00–11.50)	0.001*
FORT (%)	67.33 (50.00–77.38)	57.00 (42.50–75.00)	0.037*
Total gonadotropin dose (IU)	2650 (2193.75–3787.50)	3000 (2337.50–3750)	0.420
Oocytes retrieved	11.50 (8.00–17.00)	7.00 (5.00–11.00)	< 0.001*
Mature (MII) oocytes	10.00 (6.75–12.25)	6.00 (4.00–9.00)	< 0.001*
Available embryos	6.00 (4.00–10.00)	4.00 (2.00–5.50)	< 0.001*

Full Transfer Cohort, n = 156

*AFC* antral follicle count, *AMH* anti-Müllerian hormone, *BMI* body mass index, *FORT* follicular output rate, *PFC* pre-ovulatory follicle count

*p < 0.05 (Mann–Whitney U test)

### Multivariable predictors of clinical pregnancy

On multivariable logistic regression (Table 5), FORT as a continuous variable (OR 1.040, 95% CI 1.002–1.079; p = 0.039) and the number of available embryos (OR 1.347, 95% CI 1.082–1.676; p = 0.008) were the only parameters independently associated with clinical pregnancy after adjustment for age, BMI, AFC, AMH, PFC, total gonadotropin dose, oocyte number, and M2 oocyte number. Static ovarian reserve markers (AMH, AFC), age, and total gonadotropin dose did not reach statistical significance in the multivariable model.

**Table 5:** Multivariable logistic regression: independent predictors of clinical pregnancy

Parameter	Odds Ratio	95% CI Lower	95% CI Upper	p value
Age (years)	0.955	0.864	1.056	0.371
BMI (kg/m^2^)	1.043	0.923	1.179	0.499
AMH (ng/mL)	0.953	0.752	1.207	0.688
AFC	1.080	0.958	1.219	0.208
PFC	0.862	0.701	1.061	0.161
FORT (%–continuous)	1.040	1.002	1.079	0.039*
Total gonadotropin dose (IU)	1.0002	0.9997	1.0007	0.536
Oocytes retrieved	1.131	0.931	1.374	0.216
Mature (MII) oocytes	0.877	0.688	1.119	0.291
Available embryos	1.347	1.082	1.676	0.008*

*AFC* antral follicle count, *AMH* anti-Müllerian hormone, *BMI* body mass index, *FORT* follicular output rate, *PFC* pre-ovulatory follicle count

*p < 0.05

### Exploratory ROC analysis

Table 6 presents the results of the exploratory ROC analysis. The AUC for FORT predicting clinical pregnancy was 0.607 (95% CI 0.52–0.69). The Youden-index-derived optimal threshold was 64.2%, with sensitivity 56.3% (95% CI 42.2–69.7%) and specificity 63.9% (95% CI 53.5–73.4%). Clinical pregnancy occurred in 27 of 66 patients (40.9%) with FORT ≥ 64.2% and in 21 of 90 patients (23.3%) with FORT < 64.2% (p = 0.030; OR 2.27, 95% CI 1.14–4.55).

**Table 6:** Exploratory ROC analysis: FORT threshold for clinical pregnancy prediction

Parameter	Value	95% CI
ROC curve analysis		
Optimal FORT threshold (%)	64.2	–
Area under the curve (AUC)	0.607	0.52–0.69
Sensitivity (%)	56.3	42.2–69.7
Specificity (%)	63.9	53.5–73.4
Threshold-based stratification		
High-FORT group (≥ 64.2%), n = 66	Clinical pregnancy: 27 (40.9%)	29.0–53.7
Low-FORT group (< 64.2%), n = 90	Clinical pregnancy: 21 (23.3%)	15.0–33.1
Association measures		
Chi-square (χ^2^)	4.73	–
p value	0.030*	–
Odds ratio	2.27	1.14–4.55

*AUC* area under the curve, *FORT* follicular output rate, *ROC* receiver operating characteristic. Threshold derived by Youden's index. Results are exploratory; external validation is required before clinical implementation

*p < 0.05

The magnitude of the AUC is consistent with the multifactorial nature of ART outcomes and indicates that FORT should be interpreted as a contributory, rather than independent, predictor. The identified threshold is exploratory and intended to support hypothesis generation. It may serve as a reference point for stratified counselling and for prospective validation in future studies, but it should not be applied as a standalone clinical decision criterion.

## Discussion

This prospective cohort study demonstrates that FORT is significantly associated with embryological outcomes, clinical pregnancy, and live birth rate in non-PCOS patients undergoing IVF across two distinct stimulation protocols. Clinical pregnancy rates increased across FORT tertiles (14.9%, 27.8%, and 37.2%; p = 0.045), and FORT remained independently associated with pregnancy outcomes in multivariable analysis. These findings suggest that FORT reflects aspects of ovarian responsiveness during stimulation that are not fully captured by conventional static measures of ovarian reserve.

Our results corroborate and extend the seminal work by Genro et al., who first introduced FORT as a measure of follicular responsiveness [5]. FORT was first described by Genro et al. in 2011 as an index of follicular responsiveness in normo-cycling women undergoing long GnRH agonist stimulation [5]. In that study, FORT showed an inverse association with AMH, suggesting that a larger antral follicle pool does not necessarily translate into higher follicular efficiency during stimulation. Gallot et al. subsequently reported positive associations between FORT, fertilization rate, and embryo quality in an agonist-protocol cohort, and identified a data-driven threshold in the range of 60–70% that was linked to more favorable outcomes [6].

Additional studies, including those by Rehman et al. [10, 11], Hassan et al. [12], and Zhang et al. [13], also reported associations between higher FORT values and improved embryological parameters. However, these studies applied heterogeneous AFC definitions, with upper size limits ranging from 9 to 11 mm, which complicates direct comparison across cohorts.

In the present study, FORT was calculated according to the original definition proposed by Genro et al., with the AFC denominator restricted to follicles measuring 3–9 mm and the pre-ovulatory follicle numerator defined as follicles measuring 16–24 mm. This approach ensures methodological consistency with the founding publication and closely related studies. The threshold identified in the current cohort (64.2%) is similar to that reported by Gallot et al. and lies within the upper FORT ranges described previously, although prospective validation is required. An additional contribution of this study is the evaluation of FORT in GnRH antagonist and short flare-agonist protocols, which are now widely used, as well as the inclusion of live birth as an outcome, a measure reported in relatively few prior FORT studies.

### FORT as a complement to static ovarian reserve markers

FORT combines information on baseline ovarian reserve, represented by AFC, with the number of follicles that progress to the pre-ovulatory stage during stimulation, providing a ratio that reflects follicular efficiency and its responsiveness within a given cycle. Patients with similar AMH and AFC values may exhibit different ovarian responses to stimulation, and this variability can be quantified using FORT, which may provide prognostic information. In multivariable analysis, FORT remained independently associated with clinical pregnancy, whereas AMH and AFC did not retain significance after adjustment, despite showing associations in univariable analyses. Comparable findings have been reported by Hassan et al. in patients with unexplained infertility [12]. In addition, the follicle-to-oocyte index (FOI), which reflects a related aspect of follicular response, has been associated with reproductive outcomes in studies by Alviggi et al. [3] and Cesarano et al. [4]. Collectively, these observations indicate that cycle-specific measures of follicular responsiveness provide information that complements conventional ovarian reserve markers rather than replacing them [17–21].

The AUC of 0.607 (95% CI 0.52–0.69) for FORT in predicting clinical pregnancy indicates modest discriminatory ability. This is consistent with the multifactorial nature of IVF outcomes, which are influenced by several determinants, including endometrial receptivity, embryo quality, luteal phase support, and patient-specific factors. No single parameter is expected to fully capture this complexity. Similar AUC values have been reported for established ovarian reserve markers such as AMH and AFC, which typically show limited predictive performance for live birth in large cohort studies [1, 2].

Accordingly, the threshold of 64.2% was considered exploratory. It represents a data-derived reference point that may be useful for stratification in counselling discussions, while also serving as a hypothesis for future validation. This cutoff should be interpreted cautiously and not used as a standalone decision criterion or to guide treatment exclusion. Its clinical utility will depend on confirmation in independent prospective, preferably multicentre, cohorts before any application in routine practice. External validation studies and integration with other key performance indicators will further refine the clinical applicability of this threshold.

### PCOS subgroup: null findings and their interpretation

The lack of significant associations between FORT and outcomes in the PCOS subgroup should be interpreted with caution. Several factors may contribute to this finding. In PCOS, the AFC denominator is typically elevated due to multifollicular ovarian morphology, which may introduce denominator-related bias and attenuate FORT values. This effect was evident in the present cohort, where median AFC across FORT tertiles ranged from 23 to 40 in PCOS patients, compared with 10 to 15.5 in non-PCOS patients.

In addition, follicular recruitment patterns in PCOS differ from those observed in non-PCOS patients. Ovarian stimulation in PCOS often yields high oocyte numbers across a wide range of FORT values, suggesting that the relationship between follicular efficiency and downstream outcomes may be less informative in this population. Finally, the PCOS subgroup was small (n = 30, with 10 patients per tertile), resulting in limited statistical power. At this sample size, even clinically relevant associations between FORT and reproductive outcomes would be unlikely to reach statistical significance. Previous studies evaluating FORT in PCOS populations have reported heterogeneous results. Yang et al. [15] observed a positive association between FORT and cumulative live birth rate, whereas Tan et al. [16] reported increasing clinical pregnancy rates with higher FORT values. In contrast, Zhang et al. [13] identified optimal outcomes in the intermediate FORT group. This heterogeneity likely reflects the same methodological and population-size challenges that affected our subgroup analysis. We therefore concur with prior authors that FORT may have population-specific validity constraints in PCOS, and that dedicated, adequately powered studies in PCOS are needed.

A distinctive feature of this study's design is that all embryo transfers were conducted in HRT-FET cycles. This approach standardises endometrial preparation and enables cycle scheduling, but it does not replicate the hormonal milieu of a natural cycle. The exogenous progesterone and estradiol environment may modify endometrial receptivity in ways that interact with embryo quality, a variable that FORT partially reflects. Accordingly, the FORT-outcome associations reported here are specific to the HRT-FET context; whether they are equally robust in natural-cycle or modified natural-cycle FET, or following fresh transfer, cannot be established from these data.

The restriction to first-cycle outcomes is a deliberate methodological choice with acknowledged limitations. In patients with higher FORT who tend to have greater embryo availability, the true reproductive potential of a given stimulation cycle encompasses all subsequent frozen transfers. By analysing only the first FET, the live birth rate associated with any given FORT value is likely underestimated, particularly in the high-FORT group. Future studies should report cumulative live birth rates across all transfers as the primary endpoint.

### Clinical practice implications

Within the current evidence base, FORT is best positioned as a complementary, cycle-specific indicator that can be calculated without additional cost or procedures from routine ultrasound data already collected during standard IVF monitoring. Its value lies in personalised counselling: patients with low FORT (< 33rd percentile) may be counselled about reduced per-cycle success rates and the potential value of modified stimulation approaches in future cycles, while patients with FORT ≥ 64.2% can be reassured of a meaningfully higher probability of success relative to those below this value (40.9% vs. 23.3%, OR 2.27).

FORT may also serve as a programme-level key performance indicator (KPI) for ART units, enabling benchmarking of stimulation efficiency. Its protocol independence, demonstrated in both antagonist and flare-agonist cycles in this study, supports wider applicability across current practice paradigms. We do not advocate replacing AMH or AFC with FORT but rather incorporating it into a multi-parameter assessment that includes both static reserve and functional responsiveness. The additive value of combining FORT with existing markers should be evaluated in future work, ideally using individual patient data meta-analysis across existing FORT cohorts.

### Strengths and limitations

#### Strengths

This study has several notable strengths. It was conducted prospectively with pre-specified outcome measures and included live birth as an endpoint, which has been reported infrequently in the existing FORT literature. The analysis encompassed two commonly used stimulation protocols GnRH antagonist and short flare-agonist, thereby extending applicability beyond prior studies limited to long agonist regimens. Multivariable modelling demonstrated that FORT retained independent association with clinical pregnancy after adjustment for conventional reserve markers. In addition, this study provides the first empirically derived FORT threshold based on Youden index analysis within a prospective cohort. Finally, separate analyses of PCOS and non-PCOS patients were performed to reduce confounding related to differences in ovarian reserve characteristics.

#### Limitations

Several limitations should be considered when interpreting these findings. Outcomes were restricted to the first embryo transfer, which may underestimate cumulative reproductive potential. In addition, all transfers were performed in hormonally prepared frozen–thawed embryo transfer cycles, and the applicability of these results to natural-cycle FET or fresh transfer remains uncertain. The PCOS subgroup was small (n = 30), limiting the reliability of subgroup analyses. The ROC-derived threshold of 64.2% demonstrated modest discriminatory ability (AUC 0.607) and should therefore be regarded as exploratory, requiring external prospective validation before any clinical application. Finally, this study was conducted at a single tertiary center, which may limit the generalisability of the findings to other clinical settings.

## Conclusion

FORT is a cycle-specific functional indicator of follicular responsiveness that is associated with clinical pregnancy and live birth in non-PCOS patients undergoing IVF, across both antagonist and short flare-agonist stimulation protocols. Its independent association in multivariable analysis beyond static markers including AMH and AFC suggests that it may provide additional information as a complementary parameter in ART outcome stratification and patient counselling. An exploratory data-derived threshold of 64.2% was observed to be associated with higher odds of clinical pregnancy (approximately two-fold) compared with lower values. This threshold is presented as hypothesis-generating and requires prospective validation in independent cohorts before clinical application. FORT's utility in PCOS remains uncertain and warrants dedicated adequately powered study. Future multicenter investigations should evaluate FORT's contribution to cumulative live birth prediction and its integration with other emerging cycle-specific performance indices to fully characterise its role in personalised ART practice.

## Data Availability

The datasets used and/or analyzed during the current study are available from the corresponding author upon reasonable request.

## Declarations

### Trial registration

Clinical Trials Registry of India: CTRI/2023/10/058192.
